# The distribution of immune cells within combined hepatocellular carcinoma and cholangiocarcinoma predicts clinical outcome

**DOI:** 10.1002/ctm2.11

**Published:** 2020-04-18

**Authors:** Bo‐Hao Zheng, Jia‐Qiang Ma, Ling‐Yu Tian, Liang‐Qing Dong, Guo‐He Song, Jiao‐Men Pan, Yu‐Ming Liu, Shuai‐Xi Yang, Xiao‐Ying Wang, Xiao‐Ming Zhang, Jian Zhou, Jia Fan, Jie‐Yi Shi, Qiang Gao

**Affiliations:** ^1^ Department of Liver Surgery and Transplantation and Key Laboratory of Carcinogenesis and Cancer Invasion (Ministry of Education) Liver Cancer Institute Zhongshan Hospital Fudan University Shanghai China; ^2^ Department of General Surgery Zhongshan Hospital Fudan University Shanghai China; ^3^ The Center for Microbes Development, and Health Key Laboratory of Molecular Virology & Immunology Institute Pasteur of Shanghai Chinese Academy of Sciences/University of Chinese Academy of Sciences Shanghai China; ^4^ Institutes of Biomedical Sciences Fudan University Shanghai China

**Keywords:** liver cancer, programmed cell death 1 receptor, T lymphocytes, tumor microenvironment

## Abstract

**Background:**

This study aimed to investigate the clinical relevance of the immune microenvironment in patients with combined hepatocellular carcinoma and cholangiocarcinoma (cHCC‐ICC).

**Patients and Methods:**

The density of tumor‐infiltrating CD3^+^, CD8^+^, CD163^+^, and Foxp3^+^ immune cells, as well as Programmed cell death 1, Programmed cell death‐ligand 1, and Tumor necrosis factor receptor superfamily member 4, was measured in the peritumor liver, tumor invasive margin, and intratumor subregions of 56 cHCC‐ICC by immunohistochemistry. The immune index was established to stratify patients. Prognostic significance of immune cell subsets and immune indices was evaluated.

**Results:**

The distribution of immune cells was highly heterogeneous among different subregions of cHCC‐ICC. As compared with the hepatocellular carcinoma (HCC) component, the lower density of CD8^+^ T cells and higher intensity of Foxp3^+^ Tregs and immune checkpoints in the intrahepatic cholangiocarcinoma (ICC) component may indicate a stronger immune evasive ability of ICC. Based on clustering classification or a combination of random forest and lasso‐cox, two models of immune indices were established and both were identified as independent prognostic factors for cHCC‐ICC patients. The selected immune variables in the immune prognostic models derived from both HCC and ICC subregions, indicating that the prognosis of cHCC‐ICC patients was a complex interaction of both components.

**Conclusions:**

The immune contexture was heterogeneous among different subregions of cHCC‐ICC patients and contributed differently to patient prognosis. Immune score based on the densities of immune cells might serve as a promising prognostic predictor for cHCC‐ICC patients.

## Graphical abstract

Highlights

- The prognosis of cHCC‐ICC patients was a complex interaction of both cancer components.
- The immune evasive ability of ICC components is stronger than that of HCC components.
- Heterogeneous immunological features could serve as a promising prognostic index for cHCC‐ICC patients.
- Immunotherapy strategies could be a potential treatment for cHCC‐ICC patients.

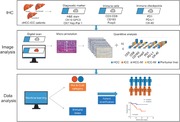

**Abbreviations**
cHCC‐ICC: combined hepatocellular carcinoma and cholangiocarcinoma
CI: confidence interval
HBV: hepatitis B virus
HCC: hepatocellular carcinoma
HR: hazard ratio
ICC: intrahepatic cholangiocarcinoma
IDH1: isocitrate dehydrogenase‐1
IM: invasion margin
OS: overall survival
OX40: Tumor necrosis factor receptor superfamily member 4
PD1: Programmed cell death 1
PD‐L1: Programmed cell death‐ligand 1
TNM: tumor‐node‐metastases

## 1 BACKGROUND

Liver cancer is the fourth leading cause of cancer‐related deaths, with more than 85 000 new cases annually worldwide.^1^ Combined hepatocellular carcinoma and cholangiocarcinoma (cHCC‐ICC), a rare type of primary liver cancer, accounts for 1‐14.2% of all primary liver malignancies.^2^ The survival of cHCC‐ICC is significantly worse than hepatocellular carcinoma (HCC) and more similar to intrahepatic cholangiocarcinoma (ICC).^3^, ^4^, ^5^, ^6^ Due to the relatively low incidence of cHCC‐ICC, the molecular pathogenesis and the clinical behavior of these tumors remain ill‐defined. To date, clinical guidelines do not propose a specific treatment recommendation for cHCC‐ICC patients. Hepatectomy remains the only curative treatment that amenable for early‐stage patients, albeit modest benefits and high recurrence rate.^2^ For those cHCC‐ICC patients in the advanced stage, standard systemic therapies are still not available. Hence, new treatment strategies are urgently needed for cHCC‐ICC patients.

Recent data have demonstrated significant benefits of immunotherapy in various solid tumors, including nonsmall cell lung cancer,^7^ genitor‐urinary cancer,^8^ HCC,^9^ and ICC.^10^ However, there is still no such ongoing clinical trials for cHCC‐ICC patients. The basic principle of immunotherapy is the modulation of tumor‐immune interactions. Several studies have reported the epigenetic, genetic, and transcriptomic signatures of cHCC‐ICC patients,^11^, ^12^, ^13^ but the understanding of the immune microenvironment in cHCC‐ICC is still lacking. Based on the density and distribution of CD3^+^ and CD8^+^ T cells, the “hot and cold” classification for the tumor was postulated, which could predict clinical outcomes of patients with various cancers and “hot” indicated potential sensitivity to immunotherapy.^14^ It is rational to speculate that a comprehensive analysis of the type, density, and spatial distribution of immune components within the local microenvironment may provide important clues for developing immunotherapy for cHCC‐ICC patients.

In this study, we carried out a preliminary quantitative and qualitative assessment of immune contexture in cHCC‐ICC patients. Immunohistochemical characterization of CD3 (Pan‐T cells), CD8 (T‐killer cells), Foxp3 (Regulatory T cells [Tregs]), and CD163 (macrophages), as well as immune checkpoints Programmed cell death 1 (PD‐1), Programmed cell death‐ligand 1 (PD‐L1), and Tumor necrosis factor receptor superfamily member 4 (OX40), was conducted in a consecutive cohort of 56 cHCC‐ICC patients. We showed a tumor subregion‐specific infiltration of immune cells that contributed differently to patient prognosis. As compared with HCC subregions, lack of CD8^+^ T cells, enriched Tregs, and a higher level of immunoinhibitory checkpoints in ICC subregions may indicate a stronger immune evasive ability of ICC.

## 2 PATIENTS AND METHODS

### 2.1 Patient selection

This retrospective analysis included 56 consecutive cHCC‐ICC patients who underwent curative resection of their primary tumors between 2011 and 2015 at Zhongshan Hospital. The criteria for the enrolled patients were listed in Additional file 1. Tumors were assigned a pathological tumor‐node‐metastases (TNM) stage according to the American Joint Committee on Cancer (AJCC) 8^th^ edition.^15^ According to the Allen and Lisa criteria,^16^ cHCC‐ICC was divided into three types: separated tumor, combined type, and mixed type (Additional file 2). This study was approved by the Institutional Review Board (B2017‐060) and was performed following the Declaration of Helsinki.

Postoperative surveillance and treatment modality was performed as we previously described.^17^ The definition of overall survival (OS) was the span from the first resection to death or censored for living patients.

### 2.2 Immunohistochemistry

Immunohistochemistry was conducted as described previously.^18^ Detail procedures were depicted in Supplementary Method. Details of antibodies used were presented in Table S1.

### 2.3 Microanatomical annotation

**FIGURE 1:**
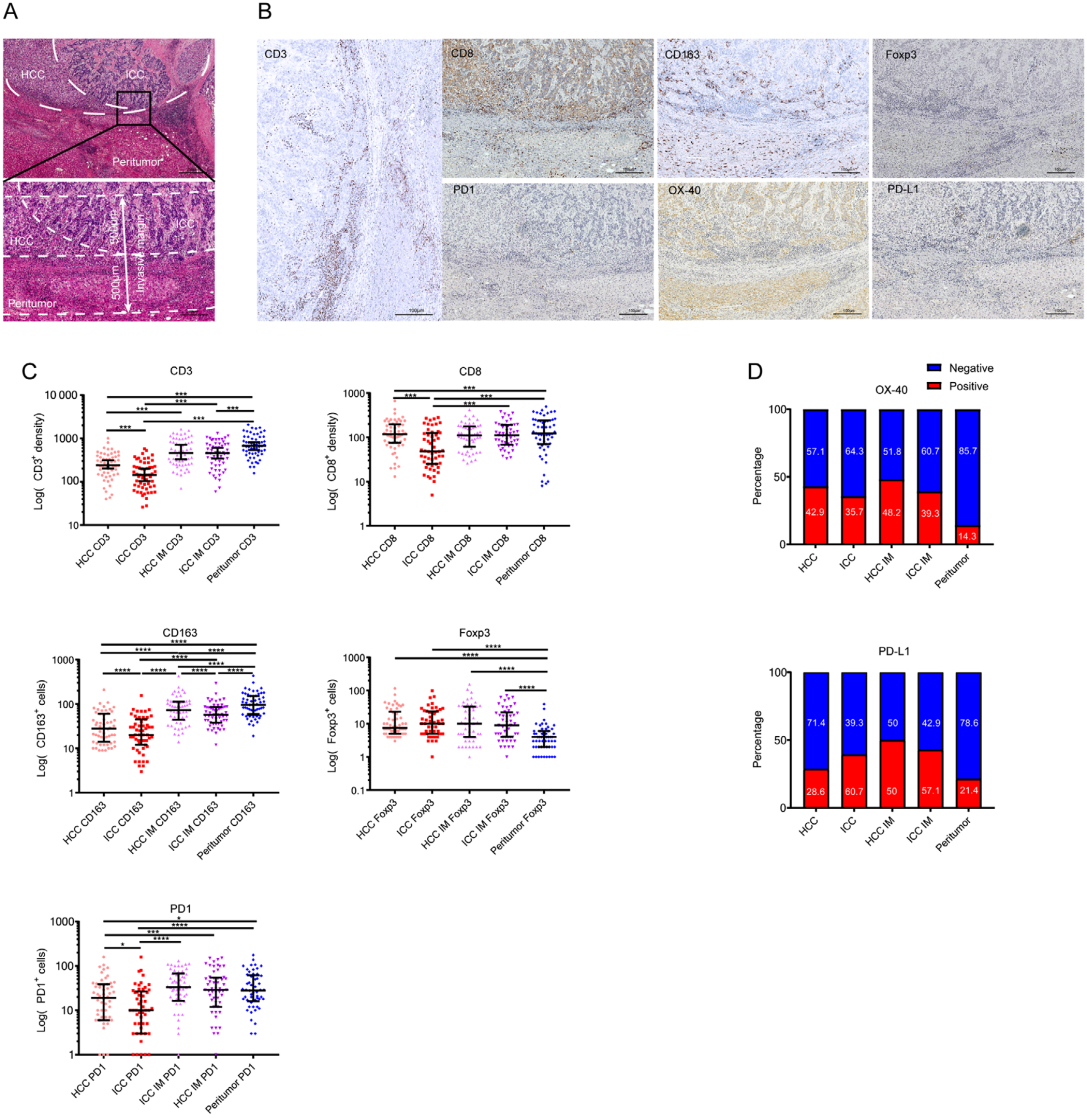
Representative staining pictures and spatial distribution of immune variables in combined hepatocellular carcinoma and cholangiocarcinoma (cHCC‐ICC). A, Tumor micro‐annotation and the definition of the tumor invasive margin (magnification, ×4 and ×100). B, The representative images of indicated immune variables in cHCC‐ICC. Positive cells were stained brown (magnification, ×100). C, Statistics depicting the spatial distribution of infiltrating immune cells (^*^ *P* < .05; ^**^ *P* < .01; ^***^ *P* < .001). D, Statistics depicting the spatial distribution of the immune checkpoints

To evaluate the spatial heterogeneity of immune components, tumor sections were microanatomically divided into intratumor, invasion margin (IM), and peritumor liver. The tumor IM was defined as the region within 500 μm on each side of the border between the tumor and normal liver tissue^19^ (Figure 1A). For separated and combined subtypes of cHCC‐ICC, the intratumor area was divided into HCC and ICC components, whereas the IM was divided into HCC IM and ICC IM. For mixed subtype, the HCC and ICC components were defined as areas where HCC and ICC cells accounting for 90% of all the tumor cells, respectively. Details of the annotation strategy were presented in Figure S2.

### 2.4 Quantification of CD3, CD8, Foxp3, and PD1 positive immune cells

The density of positive cells was evaluated as described previously.^19^ In brief, three microscopic fields (magnification, ×200) of each hot area were selected and captured. Positively stained cells were counted by using the IHC toolbox.^20^, ^21^ Then, the density of positive cells was calculated by averaging.

### 2.5 Quantification of the expression of PD‐L1 and OX40

For the immune checkpoints, a digital image system was used to evaluate the signals as described previously.^22^ Detailed information was listed in Additional file 1.

### 2.6 Clustering analysis and development of the immune score

Unsupervised clustering analysis of the densities of immune cells and immune checkpoints was performed using Euclidean distance. Random forest for survival analysis using the “*randomForestSRC*” package was used to select the most important prognostic factors. Then, the lasso‐cox using the “*glmnent*” package was implemented to establish an immune score. All analyses were carried out in R (R foundation for statistical computing, Vienna, Austria; URL: http://www.R-project.org; 2016). Then, C‐index was used to evaluate the prognostic significance of the two categories and compared via the Delong test.

### 2.7 Statistical analysis

The results were presented as mean ± standard deviation (SD) or median (range). The Fisher's exact test, Chi‐squared test, or Mann‐Whitney U test was used as appropriate. Paired Wilcoxon signed‐rank tests was used to analysis the difference in the distribution of immune cells, whereas Spearman's rank correlation analysis was performed to analysis the correlations between the immune cells. The survival curve was depicted by using Kaplan‐Meier and compared via the log‐rank test. The Cox hazard regression model was carried out for univariate and multivariate analyses. A two‐tailed *P* < .05 was considered statistical significance. SPSS 22.0 software (Chicago, IL, USA) and Graphpad Prism 7 software (La Jolla, CA, USA) were used to conduct the statistical analyses.

## 3 RESULTS

### 3.1 Baseline characteristics of patients

The characteristics of the study cohort are listed in Table 1. Among the 56 cHCC‐ICC cases, one was defined as separated type, 24 were combined type, and 31 were mixed type (Figure S1). The number of patients at TNM stages Ia, Ib, II, and IV were 34, 10, 10, and 2, respectively. The 1‐, 3‐, and 5‐year postoperative survival rates were 86%, 67%, and 57%, respectively. In our study, the 5‐year survival rate was better than previously reported ones,^15^ possibly due to that most patients were at the early stage and all patients received R0 resection. Besides, only two patients were found to have lymph node metastases.

**TABLE 1:** Clinicopathological features of combined hepatocellular carcinoma and cholangiocarcinoma (cHCC‐ICC) patients (n = 56)

Variables	
Age, median (range)	56 (29‐74)
Gender	
Male	45
Female	11
Liver cirrhosis	
Absent	13
Present	43
Max tumor size, cm	
Median (range)	3.5 (0.5‐8.0)
Tumor number	
Single	46
Multiple	10
Microvascular invasion	
Absent	44
Present	12
Lymph node metastases	
Absent	54
Present	2
Macrovascular invasion	
Absent	54
Present	2
CA19‐9, ng/mL	
Median (range)	25.6 (3.2‐254.5)
AFP, ng/mL	
Median (range)	46.7 (0.7‐60 500)
Pathological type	
Separated	1
Combined	24
Mixed	31
TNM stage	
Ia	34
Ib + II + IV	22

Abbreviations: TNM, tumor‐node‐metastasis; AFP, alpha‐fetoprotein; CA19‐9, antigen carbohydrate 19‐9.

### 3.2 Density and distribution of immune cells and checkpoints in cHCC‐ICC

Tumor slides were divided into five subregions, including the HCC component, ICC component, HCC‐IM, ICC‐IM, and the peritumor liver. Positive staining of Hep‐Par1 and GPC3 was defined as the HCC component, whereas the positivity of CK7 and CK19 was defined as the ICC component^16^, ^23^, ^24^ (Figure S1). The representative staining of immune variables (including CD3, CD8, CD163, Foxp3, PD1, PD‐L1, and OX40) using consecutive sections is presented in Figure 1B. Accordingly, the intensity of immune cells and checkpoints were evaluated at different microanatomical subregions (Figures 1C and 1D).

CD3^+^ T cells were predominately enriched in the peritumor liver (672.50/mm^2^), followed by HCC‐IM (458.50/mm^2^), ICC‐IM (456.50/mm^2^), and HCC component (239/mm^2^), with the least in ICC component (143.50/mm^2^). The distribution of CD8^+^ T cells was similar to CD3^+^ T cells, which was abundant in the peritumor liver (123/mm^2^), followed by HCC‐IM (111/mm^2^), ICC‐IM (112/mm^2^), and HCC component (103/mm^2^), and generally low in ICC component (48/mm^2^). Also, CD163^+^ macrophages were enriched in the peritumor liver (95/mm^2^); moderate in HCC‐IM (73/mm^2^) and ICC‐IM (57/mm^2^); and very low in HCC component (28/mm^2^) and ICC component (20/mm^2^). In contrast, the density of Foxp3^+^ Tregs in ICC component was the highest (10/mm^2^), followed by HCC‐IM, ICC‐IM (10/mm^2^/9/mm^2^), HCC component (7.5/mm^2^), and the peritumor liver (4/mm^2^). The density of PD‐1^+^ cells was high in HCC‐IM (33/mm^2^), intermediate in ICC‐IM (29/mm^2^) and the peritumor liver (28/mm^2^), and very low in ICC component (10/mm^2^) (Figure 1C; Table 2). These results indicated that ICC component was specifically enriched with Tregs and sparsely infiltrated with CD8^+^ T cells, as compared with other microanatomical subregions.

**TABLE 2:** Descriptive statistics of immunohistochemical variables

	CD3		CD8		CD163		Foxp3		PD1^+^ cells	
	Median		Median		Median		Median		Median	
Subregions	(cell/mm^2^)	Range	(cell/mm^2^)	Range	(cell/mm^2^)	Range	(cell/mm^2^)	Range	(cell/mm^2^)	Range
HCC component	239	40‐1011	103	13‐662	28	9‐210	7.5	3‐116	19	0‐159
ICC component	143.5	26‐573	48	5‐281	20	3‐155	10.0	1‐100	10	0‐158
HCC‐IM	458.5	70‐1627	111	26‐419	73	14‐429	10.0	1‐154	33	0‐131
ICC‐IM	456.5	59‐1314	112	33‐382	57	12‐294	9	1‐71	29	0‐152
Peritumor liver	672.5	157‐2072	123	8‐493	95	19‐434	4	1‐39	28	0‐176
*P*‐values										
HCC vs ICC	<.001		<.001		<.001		.665		.011	
HCC vs HCC‐IM	<.0001		.454		<.001		.254		.005	
ICC vs ICC‐IM	<.001		<.001		<.001		.828		.000	
HCC‐IM vs peritumor	.076		.107		.004		<.001		.655	
ICC‐IM vs peritumor	<.001		.367		<.001		<.001		.458	
HCC‐IM vs ICC IM	.925		.791		.002		.203		.808	
HCC vs peritumor	<.001		<.001		<.001		<.001		.029	
ICC vs peritumor	<.001		<.001		<.001		<.001		<.001	

*Note*. Wilcoxon signed‐rank test.

Then, the expression patterns of OX40 and PD‐L1 were analyzed (Figure 1D; Table 2). We found that the positive rate of OX40 was similar between each tumor subregion and their corresponding IM (HCC: 42.9% vs HCC‐IM: 48.2%, *P* = .285; ICC: 35.7% vs ICC‐IM: 39.3%, *P* = .789), but lower in the peritumor liver (14.3%). Of importance, the positive rate of OX40 in HCC component was slightly higher than that in ICC component (HCC: 42.9% vs ICC: 35.7%, *P* = .033). For PD‐L1, the positive rate was higher in IM, with comparable levels between HCC‐IM and ICC‐IM (HCC‐IM: 50.00% vs ICC‐IM: 42.86%, *P = *.345). In contrast, the positivity of PD‐L1 in ICC component was significantly higher than that in HCC component (ICC: 39.29% vs HCC: 28.57%, *P *= .002). Taken together, all these data showed obvious spatial heterogeneity of immune contexture in the cHCC‐ICC microenvironment. The lower density of CD8^+^ T cells and higher intensity of immune checkpoints in the ICC component and ICC invasive margin may indicate a stronger immune evasive ability of ICC.

### 3.3 The prognostic values of immune cells and checkpoints

Due to their heterogeneous distribution, the prognostic values of immune variables in different subregions were analyzed individually. Patients were stratified into high and low groups based on the optimal cutoff of each immunostaining variable determined by cutoff finder.^25^

For T cells, patients with high density of CD3^+^ or CD8^+^ T cells were associated with better survival, including HCC component, ICC‐IM, or peritumor liver. For CD163^+^ macrophages or Foxp3^+^ Tregs, patients with high density in HCC or ICC components were associated with worse survival. Likewise, patients with high PD1^+^ immune cells in HCC component, HCC‐IM, or ICC‐IM were associated with worse survival. No prognostic significances were observed for OX‐40 expression across all subregions, whereas patients with positive PD‐L1 in HCC or ICC components were associated with worse survival (Table S2).

Then, multivariate analyses identified that CD3 in HCC component (hazard ratio [HR] = 0.299; 95% confidence interval [CI], 0.111‐0.807; *P *= .017) and ICC‐IM (HR = 0.344; 95% CI, 0.134‐0.886; *P *= .027), CD8 in HCC component (HR = 0.234; 95% CI, 0.092‐0.592; *P* = .002), ICC‐IM (HR = 0.375; 95% CI, 0.142‐0.992; *P *= .048), peritumor liver (HR = 0.254; 95% CI, 0.092‐0.705; *P *= .009), Foxp3 in ICC component (HR = 3.426; 95% CI, 1.328‐8.841; *P *= .011), PD‐1 in HCC‐IM (HR = 0.239; 95% CI; 0.085‐0.672; *P *= .007), PD‐L1 in HCC (HR = 3.132; 95% CI, 1.258‐7.796; *P *= .014), and ICC components (HR = 3.844; 95% CI, 1.419‐10.414; *P *= .008) were independent prognostic factors for OS in cHCC‐ICC patients (Table 3). The survival analysis indicated that the prognostic significance of immune variables varied among different tumor subregions. Thus, we assumed that a panel of immune variables, including immune cell density and spatial distribution, should be identified and integrated to stratify patient prognosis.

**TABLE 3:** Multivariable cox proportional hazards models for overall survival

	Multivariable analysis		
Variables	HR	95%CI	*P*‐value
HCC CD3 (high vs low)	0.299	0.111‐0.807	.017
ICC‐IM CD3 (high vs low)	0.344	0.134‐0.886	.027
Peritumor CD3 (high vs low)	–	–	.064
HCC CD8 (high vs low)	0.234	0.092‐0.592	.002
HCC‐IM CD8 (high vs low)	–	–	.089
ICC‐IM CD8 (high vs low)	0.375	0.142‐0.992	.048
Peritumor CD8 (high vs low)	0.254	0.092‐0.705	.009
HCC CD163 (high vs low)	–	–	.545
ICC CD163 (high vs low)	–	–	.381
HCC‐IM CD163 (high vs low)	–	–	.093
HCC Foxp3 (high vs low)	–	–	.218
ICC Foxp3 (high vs low)	3.426	1.328‐8.841	.011
HCC PD‐1 (high vs low)	–	–	.161
HCC‐IM PD1 (high vs low)	0.239	0.085‐0.672	.007
ICC‐IM PD1 (high vs low)	–	–	.065
HCC PD‐L1 (positive vs negative)	3.132	1.258‐7.796	.014
ICC PD‐L1 (positive vs negative)	3.844	1.419‐10.414	.008
Cluster (2 vs 1)	4.191	1.005‐18.253	.023
Immune score (high vs low)	29.266	8.157‐105.00	<.001

Abbreviation: IM, invasive margin.

### 3.4 Patient classification based on unsupervised clustering of immune variables

**FIGURE 2:**
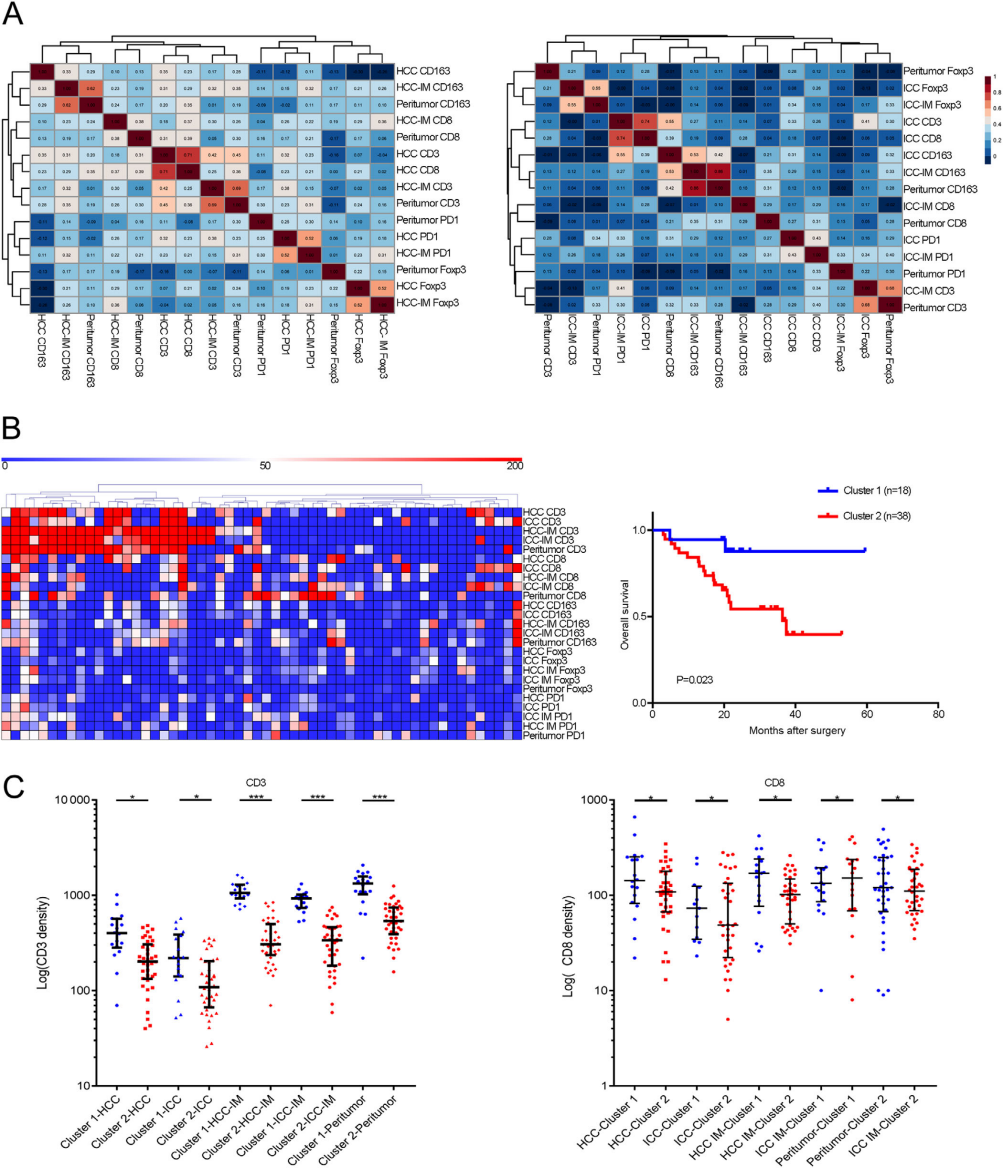
The correlation between the immune cells and the cluster based on the density of immune cells. A, The correlation between the immune cells among different subareas (hepatocellular carcinoma [HCC] [H]; intrahepatic cholangiocarcinoma [ICC] [I]; HCC invasive margin [H.IM]; ICC invasive margin [I.IM]; peritumor [P]). B, The clustering classification based on the density of the immune variables. The green bar means cluster 1 (n = 18), whereas the red bar means the cluster 2 (n = 38). Kaplan‐Meier curve of overall survival indicated that patients in cluster 1 (n = 18) correlated with better outcomes (*P* = .023). C, Patients in cluster 1 were abundant in CD3^+^ and CD8^+^ T cells in HCC, ICC, HCC invasive margin, ICC invasive margin, and peritumor areas. ^*^ *P* < .05; ^**^ *P* < .01; ^***^ *P* < .001

The correlations between immune variables were analyzed (Figure 2A). The densities of most tumor‐infiltrating immune subsets significantly and positively correlated with each other (range of correlation coefficients, 0.237‐0.686; *P *= .007 to <.001 for significant correlations).

**FIGURE 3:**
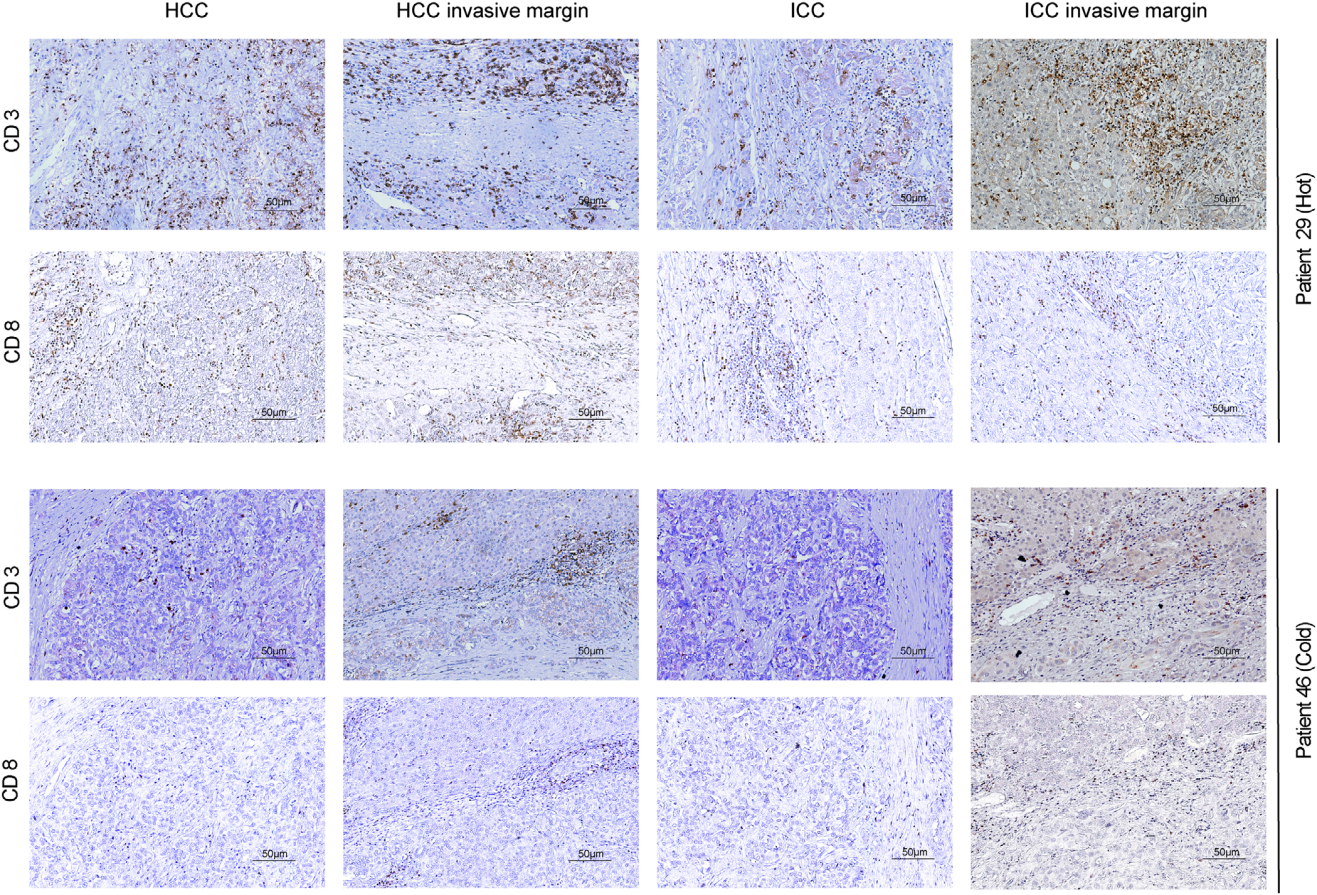
The representative images of the cold and hot tumors. A, A representative case of a hot tumor with abundant CD3^+^ and CD8^+^ immune cells (magnification, ×200). B, A representative case of a cold tumor with sparse CD3^+^ and CD8^+^ immune cells (magnification, ×200)

Several recent studies have demonstrated that tumors could be divided into different subtypes according to the density and distribution of immune cells.^26^, ^27^ Herein, unsupervised clustering based on the immune density identified two subgroups with significantly different survival, where cluster 1 had a significantly better OS than cluster 2 (*P *= .023) (Figure 2B). Multivariate analysis further identified this clustering as an independent prognostic factor for cHCC‐ICC patients (HR = 4.191; 95% CI, 1.005‐18.253; *P* = .023; Table 3). Patients in cluster 2 were associated with larger tumor size (*P *= .029), advanced TNM stage (*P *= .002), and higher hepatitis B virus (HBV) infection rate (*P *= .021) (Additional file 6). Notably, patients in cluster 1 were more abundant in CD3^+^ and CD8^+^ T cells among all subregions (Figure 2C), whereas no significant differences were observed for the densities of CD163^+^ macrophages and Foxp3^+^ Tregs. Interestingly, this clustering was consistent with the classical concept of immune “hot and cold.”^23^, ^28^, ^29^ The typical cases of hot and cold tumors were presented in Figure 3.

### 3.5 Establishment and prognostic significance of an immune index based on lasso‐cox

**FIGURE 4:**
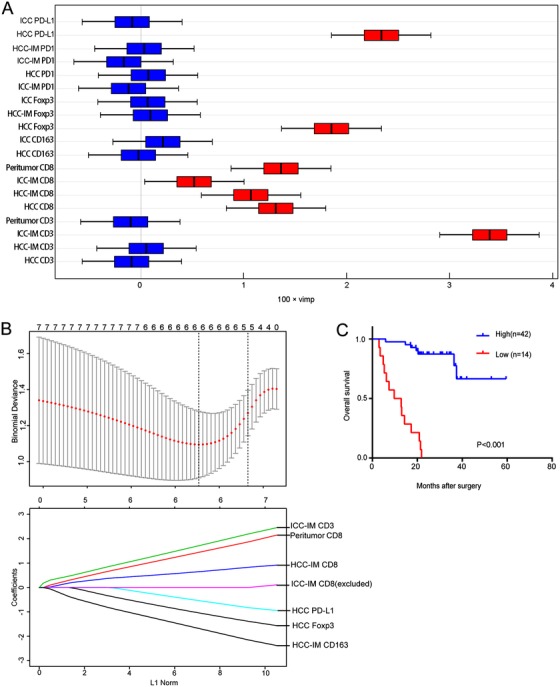
The establishment and the prognostic significance of the immune score. A, The results of the random forest for selecting variables related to survival. Seven parameters (the red ones) were selected out by the Random forest. B, Six parameters were selected out by the lasso‐cox to establish the immune score. C, The Kaplan‐Meier curve of overall survival showed that patients with high immune score were associated with better overall survival (*P* < .001)

Although the immune clustering could predict the postoperative survival of cHCC‐ICC, it is unable to predict for linear measurement of risk. Thus, we utilized the random forest to select factors that contributed most to patient prognosis. Accordingly, the expression of PD‐L1 in HCC, the densities of CD3^+^ T cells and CD163^+^ macrophages in HCC‐IM, the densities of CD8^+^ T cells in the peritumor liver, HCC‐IM, and ICC‐IM, and the density of Foxp3^+^ Tregs in the HCC components were selected out (Figure 4A). Then, lasso‐cox was used to establish the immune index based on the selected immune variables (Figure 4B), using the following formula: –2.7640938 −0.9704039*PD‐L1^HCC^ −1.3569411*Foxp3^HCC^ −1.67459*CD163^HCC‐IM^ + 0.6143227*CD8^peritumor^ + 0.4881430*CD8^HCC‐IM^ + 1.5869789*CD3^ICC‐IM^. Notably, patients with a low immune score had a significantly worse survival than those with a high immune score (*P *< .001; Figure 4C). The selected immune variables in this score derived from both HCC and ICC subregions, indicating that the prognosis of cHCC‐ICC patients was a complex interaction of both components. Multivariate analysis further identified this immune score as an independent prognostic factor for cHCC‐ICC patients (HR = 0.034; 95% CI, 0.010‐0.123; *P *< .001; Table 3). Furthermore, the immune score had significantly higher C‐index than the clustering classification (0.850 vs 0.672; *P *< .001), indicating the superiority of the immune score for prognostic stratification of cHCC‐ICC patients.

Furthermore, we found that patients with a high immune score were positively associated with smaller tumor diameter (*P *= .003), early TNM stage (*P *< .001), and lower HBV infection rate (*P *= .037) Table S3. Although it has been reported that the immune microenvironment varied with the pathological subtypes of cHCC‐ICC,^16^ no correlation was found between cHCC‐ICC subtypes and the immune index (*P* = .612). All these results indicated that patients with a low immune score had more aggressive tumors, authenticating that immune surveillance played a critical role in the clinical outcome of cHCC‐ICC patients, irrespective of its pathological subtypes.

## 4 DISCUSSION

Growing evidence has suggested that the type, density, and location of immune cells within the local milieu may strongly influence tumor evolution and patient prognosis.^29^ In this study, we carried out a preliminary investigation on the spatial distribution and composition of the immune contexture in cHCC‐ICC patients, finding that the density of each type of immune cell varied among different tumor subregions and contributed differently to patient prognosis.

We found that the densities of CD3^+^ and CD8^+^ T cells in the HCC component were higher than those in the ICC component, whereas the density of Tregs in ICC component was higher than that in HCC component. These findings may indicate that ICC was more likely to be immune suppressed than HCC, possibly imprinting the different genetic profiles of HCC and ICC. Recently, several studies have reported the genetic profiling of HCC^30^, ^31^, ^32^ and ICC,^33^ finding distinct gene mutation patterns between the two types of liver cancer. For instance, isocitrate dehydrogenase‐1 (IDH1) mutation has been reported as a frequent mutation in ICC, occurring in 10‐20% of patients, whereas this mutation was rarely found in HCC. Recently, Gary and colleagues found that IDH1 mutations could suppress STAT1 signaling and CD8^+^ T cell accumulation to promote the immune evasion ability of gliomas.^34^ This may partly explain why the immune microenvironment of ICC was more likely to be immune suppressed than that of HCC.

As is well known, tumor immune microenvironment is spatially heterogeneous, especial between tumor core and invasive margin. Previous studies in HCC have reported that B cells, T cells, and monocytes were enriched in the invasive margin and correlated with patient prognosis.^19^, ^35^, ^36^ In colorectal cancer, the heterogeneous density and location of T cell subsets in the tumor microenvironment have also been identified as a superior prognostic factor to the traditional TNM stage.^37^ Consistent with these previous studies, distinct infiltration of immune subsets within each subregion was observed and contributed differentially to the prognosis of cHCC‐ICC patients, suggesting that the subregion‐specific immune enrichment was a promising prognostic factor for cHCC‐ICC patients. Besides, it has been demonstrated that immune cell density may affect the response to immunotherapy,^38^ as exemplified by the findings that PD1^+^ cell was a potential biomarker for anti‐PD‐1‐immunotherapy in head and neck cancer,^39^ HCC,^40^ and ICC.^41^ Of note, we found that the distribution of PD1^+^ cells was highly heterogeneous among HCC and ICC subregions, which may result in different responses to PD1 blockade. This assumption was consistent with a recent report that showed heterogeneous immune microenvironment among liver, lung, and peritoneum metastases from the same colorectal cancer patients, leading to either sensitivity or resistance to immunotherapy of each metastasis.^42^

In our study, the immune cluster based on the density of immune cells indicated that patients were able to be divided into two subgroups with the different distribution of immune cells. The cluster 1 was abundant in CD3^+^ and CD8^+^ T cells across all tumor subregions, whereas the cluster 2 was relatively in shortage of both T‐cell subsets across all subregions. This distribution fitted the classical “hot and cold” category.^28^, ^43^, ^44^ Meanwhile, an immune score was established by the lasso‐cox method that selected immune variables from both HCC and ICC subregions, indicating that the prognosis of cHCC‐ICC patients was a complex interaction of both components. However, this immune score was derived from a mathematic algorithm, and its biological significance still needs further investigation. Further prospective analysis with a larger cohort of cHCC‐ICC patients and evaluating more immune subsets such as B cells and dendritic cells are needed for an in‐depth understanding of the clinical relevance of heterogeneous immune microenvironment in this fetal malignancy.

## 5 CONCLUSION

In conclusion, our results demonstrated the spatial heterogeneity of immune microenvironment in cHCC‐ICC, possibly due to the distinct genetic background of the two types of liver cancer. Prognostic immune indices were established based on the density of immune cells in HCC and ICC components, indicating that the prognosis of cHCC‐ICC patients was a complex interaction of both cancer components.

## CONFLICT OF INTEREST

The authors declare no conflict of interest.

## FUNDING INFORMATION

This work was supported by Natural Science Foundation of China (No. 91859105, 8196112802, and 81872321), Basic Research Project from Technology Commission of Shanghai Municipality (No. 17JC1402200) and Shanghai Municipal Key Clinical Specialty.

## AUTHOR CONTRIBUTIONS

QG, JYS, and BHZ were associated with conception and design of the study. BHZ, JQM, and LYT performed the experiments. QG, JYS, BHZ, and JQM drafted the manuscript. BHZ, LQD, GHS, JMP, YML, and SXY drafted and interpreted the data. XYW, JZ, JF, XMZ, JYS, and QG reviewed and edited the manuscript. All authors approved the manuscript.

## Supporting information

Supporting information

Supporting information

Supporting information

Supporting information

Supporting information

Supporting information

## Data Availability

Data are available upon reasonable request. The datasets used and/or analyzed during the current study are available from the corresponding author on reasonable request.

## References

[1] Bray F , Ferlay J , Soerjomataram I , Siegel RL , Torre LA , Jemal A . Global cancer statistics 2018: GLOBOCAN estimates of incidence and mortality worldwide for 36 cancers in 185 countries. CA Cancer J Clin. 2018;68:394‐424. 30207593 10.3322/caac.21492

[2] Brunt E , Aishima S , Clavien PA , et al. cHCC‐CCA: consensus terminology for primary liver carcinomas with both hepatocytic and cholangiocytic differentation. Hepatology. 2018;68:113‐126. 29360137 10.1002/hep.29789 PMC6340292

[3] Lee CH , Hsieh SY , Chang CJ , Lin YJ . Comparison of clinical characteristics of combined hepatocellular‐cholangiocarcinoma and other primary liver cancers. J Gastroenterol Hepatol. 2013;28:122‐127. 23034166 10.1111/j.1440-1746.2012.07289.x

[4] Lee JH , Chung GE , Yu SJ , et al. Long‐term prognosis of combined hepatocellular and cholangiocarcinoma after curative resection comparison with hepatocellular carcinoma and cholangiocarcinoma. J Clin Gastroenterol. 2011;45:69‐75. 20142755 10.1097/MCG.0b013e3181ce5dfa

[5] Vilchez V , Shah MB , Daily MF , et al. Long‐term outcome of patients undergoing liver transplantation for mixed hepatocellular carcinoma and cholangiocarcinoma: an analysis of the UNOS database. HPB. 2016;18:29‐34. 26776848 10.1016/j.hpb.2015.10.001 PMC4750226

[6] Yin X , Zhang BH , Qiu SJ , et al. Combined hepatocellular carcinoma and cholangiocarcinoma: clinical features, treatment modalities, and prognosis. Ann Surg Oncol. 2012;19:2869‐2876. 22451237 10.1245/s10434-012-2328-0

[7] Arbour KC , Riely GJ . Systemic therapy for locally advanced and metastatic non‐small cell lung cancer: a review. JAMA. 2019;322:764‐774. 31454018 10.1001/jama.2019.11058

[8] Pignot G , Loriot Y , Kamat AM , Shariat SF , Plimack ER . Effect of immunotherapy on local treatment of genitourinary malignancies. Eur Urol Oncol. 2019;2:355‐364. 31277773 10.1016/j.euo.2019.01.002

[9] El‐Khoueiry AB , Sangro B , Yau T , et al. Nivolumab in patients with advanced hepatocellular carcinoma (CheckMate 040): an open‐label, non‐comparative, phase 1/2 dose escalation and expansion trial. Lancet. 2017;389:2492‐2502. 28434648 10.1016/S0140-6736(17)31046-2 PMC7539326

[10] Kelley RK , Bridgewater J , Gores GJ , Zhu AX . Systemic therapies for intrahepatic cholangiocarcinoma. J Hepatol. 2020;72:353‐363. 31954497 10.1016/j.jhep.2019.10.009

[11] Xue R , Chen L , Zhang C , et al. Genomic and Transcriptomic profiling of combined hepatocellular and intrahepatic cholangiocarcinoma reveals distinct molecular subtypes. Cancer Cell. 2019;35:932‐947.e8. 31130341 10.1016/j.ccell.2019.04.007 PMC8317046

[12] Joseph NM , Tsokos CG , Umetsu SE , et al. enomic profiling of combined hepatocellular‐cholangiocarcinoma reveals similar genetics to hepatocellular carcinoma. J Pathol. 2019;248:164‐178. 30690729 10.1002/path.5243

[13] Moeini A , Sia D , Zhang Z , et al. Mixed hepatocellular cholangiocarcinoma tumors: cholangiolocellular carcinoma is a distinct molecular entity. J Hepatol. 2017;66:952‐961. 28126467 10.1016/j.jhep.2017.01.010

[14] Taube JM . Unleashing the immune system: PD‐1 and PD‐Ls in the pre‐treatment tumor microenvironment and correlation with response to PD‐1/PD‐L1 blockade. Oncoimmunology. 2014;3:e963413. 25914862 10.4161/21624011.2014.963413 PMC4292419

[15] Ramai D , Ofosu A , Lai JK , Reddy M , Adler DG . Combined hepatocellular cholangiocarcinoma: a population‐based retrospective study. Am J Gastroenterol. 2019;114:1496‐1501. 31335362 10.14309/ajg.0000000000000326

[16] Allen RA , Lisa JR . Combined liver cell and bile duct carcinoma. Am J Pathol. 1949;25:647‐655. 18152860 PMC1942823

[17] Zheng BH , Yang LX , Sun QM , et al. A new preoperative prognostic system combining CRP and CA199 for patients with intrahepatic cholangiocarcinoma. Clin Transl Gastroenterol. 2017;8:e118. 28981082 10.1038/ctg.2017.45 PMC5666116

[18] Gao Q , Qiu SJ , Fan J , et al. Intratumoral balance of regulatory and cytotoxic T cells is associated with prognosis of hepatocellular carcinoma after resection. J Clin Oncol. 2007;25:2586‐2593. 17577038 10.1200/JCO.2006.09.4565

[19] Shi JY , Gao Q , Wang ZC , Zhou J , et al. Margin‐infiltrating CD20(+) B cells display an atypical memory phenotype and correlate with favorable prognosis in hepatocellular carcinoma. Clin Cancer Res. 2013;19:5994‐6005. 24056784 10.1158/1078-0432.CCR-12-3497

[20] Lauridsen HM , Pellowe AS , Ramanathan A , et al. Tumor necrosis factor‐alpha and IL‐17A activation induces pericyte‐mediated basement membrane remodeling in human neutrophilic dermatoses. Am J Pathol. 2017;187:1893‐1906. 28609645 10.1016/j.ajpath.2017.04.008 PMC5530916

[21] Schneider CA , Rasband WS , Eliceiri KW . NIH image to ImageJ: 25 years of image analysis. Nat Methods. 2012;9:671‐675. 22930834 10.1038/nmeth.2089 PMC5554542

[22] Gao Q , Wang XY , Qiu SJ , et al. Overexpression of PD‐L1 significantly associates with tumor aggressiveness and postoperative recurrence in human hepatocellular carcinoma. Clin Cancer Res. 2009;15:971‐979. 19188168 10.1158/1078-0432.CCR-08-1608

[23] Chu PG , Ishizawa S , Wu E , Weiss LM . Hepatocyte antigen as a marker of hepatocellular carcinoma: an immunohistochemical comparison to carcinoembryonic antigen, CD10, and alpha‐fetoprotein. Am J Surg Pathol. 2002;26:978‐988. 12170084 10.1097/00000478-200208000-00002

[24] Tickoo SK , Zee SY , Obiekwe S , et al. Combined hepatocellular‐cholangiocarcinoma: a histopathologic, immunohistochemical, and in situ hybridization study. Am J Surg Pathol. 2002;26:989‐997. 12170085 10.1097/00000478-200208000-00003

[25] Capone M , Giannarelli D , Mallardo D , et al. Baseline neutrophil‐to‐lymphocyte ratio (NLR) and derived NLR could predict overall survival in patients with advanced melanoma treated with nivolumab. J Immunother Cancer. 2018;6:74. 30012216 10.1186/s40425-018-0383-1 PMC6048712

[26] Kang HJ , Oh JH , Chun SM , et al. Immunogenomic landscape of hepatocellular carcinoma with immune cell stroma and EBV‐positive tumor‐infiltrating lymphocytes. J Hepatol. 2019;71:91‐103. 30930222 10.1016/j.jhep.2019.03.018

[27] Li B , Cui Y , Nambiar DK , Sunwoo JB , Li R . The immune subtypes and landscape of squamous cell carcinoma. Clin Cancer Res. 2019;25:3528‐3537. 30833271 10.1158/1078-0432.CCR-18-4085 PMC6571041

[28] Pages F , Mlecnik B , Marliot F , et al. International validation of the consensus immunoscore for the classification of colon cancer: a prognostic and accuracy study. Lancet. 2018;391:2128‐2139. 29754777 10.1016/S0140-6736(18)30789-X

[29] Galon J , Bruni D . Approaches to treat immune hot, altered and cold tumours with combination immunotherapies. Nat Rev Drug Discov. 2019;18:197‐218. 30610226 10.1038/s41573-018-0007-y

[30] Calderaro J , Ziol M , Paradis V , Zucman‐Rossi J . Molecular and histological correlations in liver cancer. J Hepatol. 2019;71:616‐630. 31195064 10.1016/j.jhep.2019.06.001

[31] Nault JC , Martin Y , Caruso S , et al. Clinical impact of genomic diversity from early to advanced hepatocellular carcinoma. Hepatology. 2020;71:164‐182. 31206197 10.1002/hep.30811

[32] Zucman‐Rossi J , Villanueva A , Nault JC , Llovet JM . Genetic landscape and biomarkers of hepatocellular carcinoma. Gastroenterology. 2015;149:1226‐1239. e1224. 26099527 10.1053/j.gastro.2015.05.061

[33] Xie D , Ren Z , Fan J , Gao Q . Genetic profiling of intrahepatic cholangiocarcinoma and its clinical implication in targeted therapy. Am J Cancer Res. 2016;6:577‐586. 27152236 PMC4851838

[34] Kohanbash G , Carrera DA , Shrivastav S , et al. Isocitrate dehydrogenase mutations suppress STAT1 and CD8+ T cell accumulation in gliomas. J Clin Invest. 2017;127:1425‐1437. 28319047 10.1172/JCI90644 PMC5373859

[35] Gabrielson A , Wu Y , Wang H , et al. Intratumoral CD3 and CD8 T‐cell densities associated with relapse‐free survival in HCC. Cancer Immunol Res. 2016;4:419‐430. 26968206 10.1158/2326-6066.CIR-15-0110 PMC5303359

[36] Liu LZ , Zhang Z , Zheng BH , et al. CCL15 recruits suppressive monocytes to facilitate immune escape and disease progression in hepatocellular carcinoma. Hepatology. 2019;69:143‐159. 30070719 10.1002/hep.30134

[37] Galon J , Costes A , Sanchez‐Cabo F , et al. Type, density, and location of immune cells within human colorectal tumors predict clinical outcome. Science. 2006;313:1960‐1964. 17008531 10.1126/science.1129139

[38] Maleki Vareki S . High and low mutational burden tumors versus immunologically hot and cold tumors and response to immune checkpoint inhibitors. J Immunother Cancer. 2018;6:157. 30587233 10.1186/s40425-018-0479-7 PMC6307306

[39] Kansy BA , Concha‐Benavente F , Srivastava RM , et al. PD‐1 status in CD8(+) T cells associates with survival and anti‐PD‐1 therapeutic outcomes in head and neck cancer. Cancer Res. 2017;77:6353‐6364. 28904066 10.1158/0008-5472.CAN-16-3167 PMC5690836

[40] Ma J , Zheng B , Goswami S , et al. PD1(Hi) CD8(+) T cells correlate with exhausted signature and poor clinical outcome in hepatocellular carcinoma. J Immunother Cancer. 2019;7:331. 31783783 10.1186/s40425-019-0814-7 PMC6884778

[41] Lu JC , Zeng HY , Sun QM , et al. Distinct PD‐L1/PD1 profiles and clinical implications in intrahepatic cholangiocarcinoma patients with different risk factors. Theranostics. 2019;9:4678‐4687. 31367249 10.7150/thno.36276 PMC6643449

[42] Angelova M , Mlecnik B , Vasaturo A , et al. Evolution of metastases in space and time under immune selection. Cell. 2018;175:751‐765.e16. 30318143 10.1016/j.cell.2018.09.018

[43] Mlecnik B , Tosolini M , Kirilovsky A , et al. Histopathologic‐based prognostic factors of colorectal cancers are associated with the state of the local immune reaction. J Clin Oncol. 2011;29:610‐618. 21245428 10.1200/JCO.2010.30.5425

[44] Nearchou IP , Lillard K , Gavriel CG , Ueno H , Harrison DJ , Caie PD . Automated analysis of lymphocytic infiltration, tumor budding, and their spatial relationship improves prognostic accuracy in colorectal cancer. Cancer Immunol Res. 2019;7:609‐620. 30846441 10.1158/2326-6066.CIR-18-0377

