# Supplementary material for: The distribution of immune cells within combined hepatocellular carcinoma and cholangiocarcinoma predicts clinical outcome
Source: Clin Transl Med. 2020 Apr 18;10(1):45–56. doi: 10.1002/ctm2.11 (PMC7239312; doi:10.1002/ctm2.11)
Supplement: Supplementary file 1 — Supporting information [file CTM2-10-45-s001.docx]

**Supplementary Method**

**The criteria for the enrolled patients:**

(1) no anti-cancer treatments before surgery; (2) pathologically confirmed cHCC-ICC (3) no history of other malignancy; (4) complete removal of macroscopic tumors and negative resection margin proved by pathological examination; (5) complete clinicopathological and follow-up data.

**Immunohistochemistry**

Briefly, consecutive 4 μm-thick sections were cut from each block. After deparaffinization and rehydration, antigen retrieval was performed using EDTA buffer (PH 9.0) or citrate sodium (PH 6.0) in a microwave. After blocking endogenous peroxidase activity in 0.3% H_2_O_2_, nonspecific binding sites were blocked with goat serum, followed by serial incubation with primary and secondary antibodies. Finally, the sections were developed in diaminobenzidine solution under a microscope and counterstained with hematoxylin. Negative control slides omitting the primary antibodies were included in all assays. Consecutive sections were used for each immunostaining variable.

**Quantification of the expression of PD-L1 and OX40**

Briefly, 3 images fields (the same fields with the immune cells, magnification, ×200) of different areas were captured and saved as TIFF files. Images were analyzed with Image-Pro Plus version 6.2 software (Media Cybernetics) using a special function called the measurement of integrated absorbance, which evaluates both the area and the intensity of the positive staining. With this function, integrated absorbance of all the positive staining of immune checkpoints in each photograph was measured and its ratio to the total area of each photograph was calculated as the density of each immune checkpoint. The average integrated absorbance value (integrated absorbance/total area) on each slide (three images) was used to represent each sample.
