# Supplementary figures and images for: The distribution of immune cells within combined hepatocellular carcinoma and cholangiocarcinoma predicts clinical outcome
Source: Clin Transl Med. 2020 Apr 18;10(1):45–56. doi: 10.1002/ctm2.11 (PMC7239312; doi:10.1002/ctm2.11)

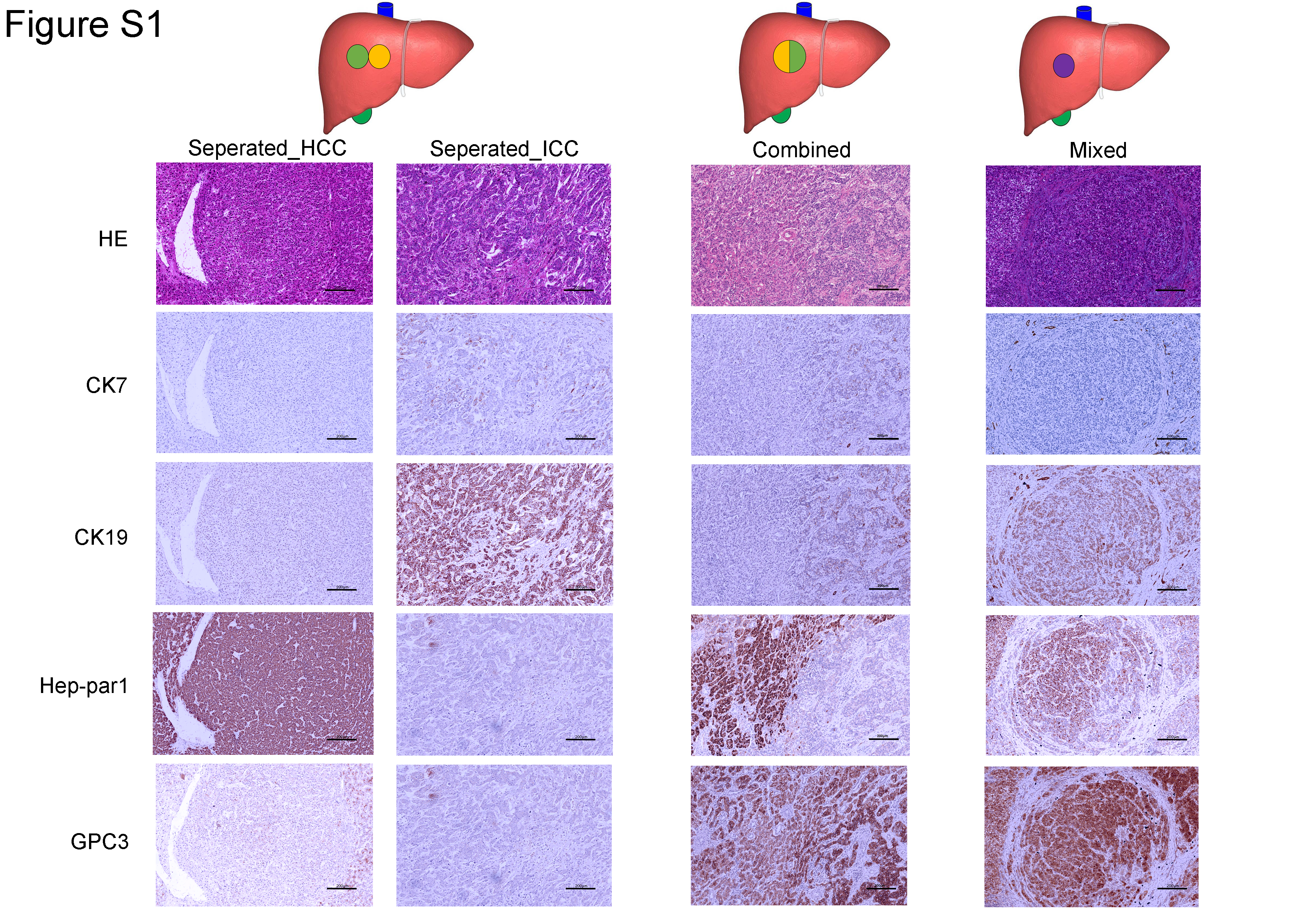

Supplement: Supplementary file 2 — Supporting information [file CTM2-10-45-s002.tif]

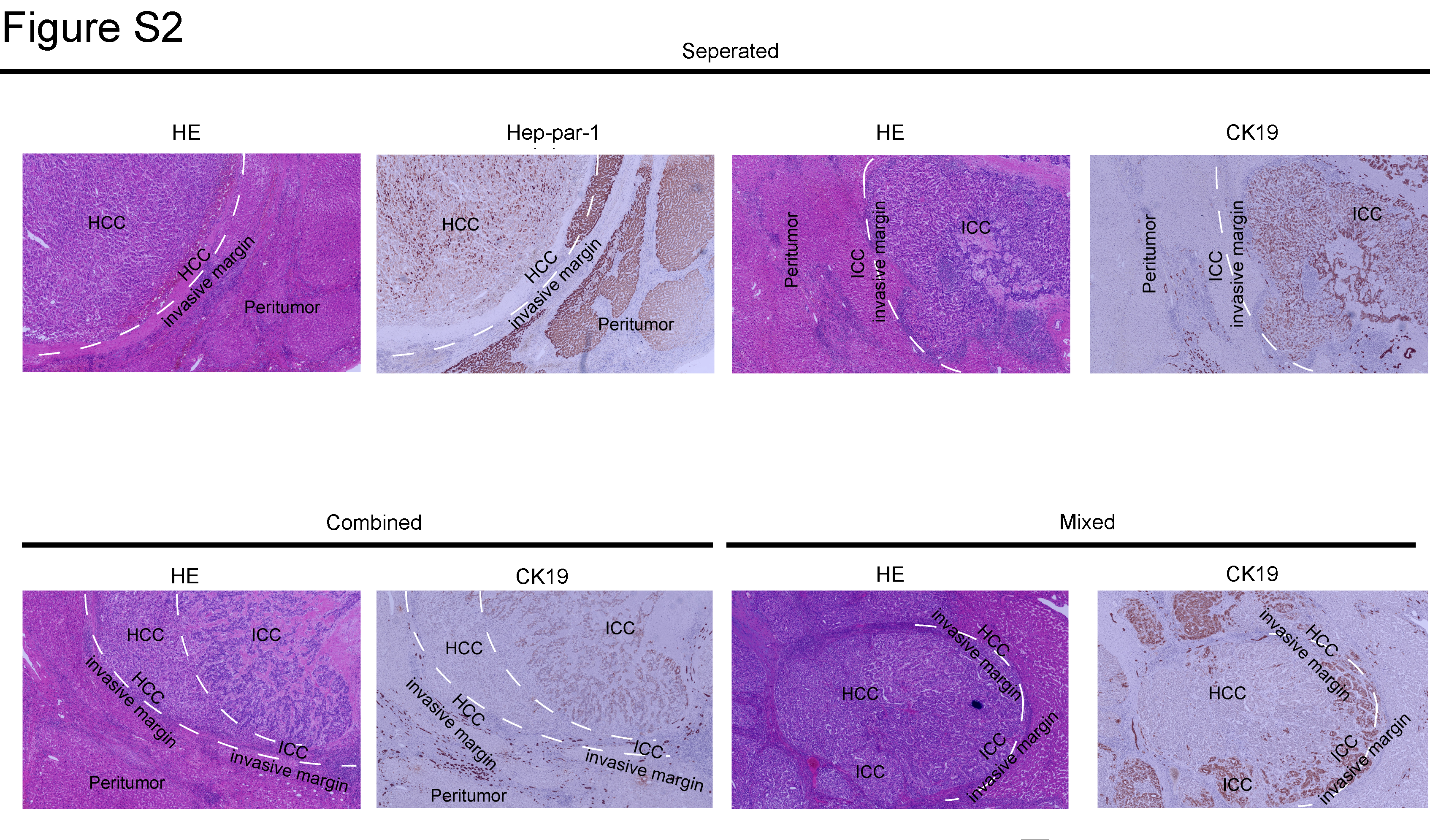

Supplement: Supplementary file 3 — Supporting information [file CTM2-10-45-s003.tif]
