# Supplementary material for: The distribution of immune cells within combined hepatocellular carcinoma and cholangiocarcinoma predicts clinical outcome
Source: Clin Transl Med. 2020 Apr 18;10(1):45–56. doi: 10.1002/ctm2.11 (PMC7239312; doi:10.1002/ctm2.11)
Supplement: Supplementary file 4 — Supporting information [file CTM2-10-45-s004.docx]

**Table S1 Primary antibodies used for immunohistochemistry**

| **Antibodies** | **Concentration** | **Specificity** | **Company** |
| --- | --- | --- | --- |
| CD3 | 1:100 | Rabbit monoclonal | Abcam |
| CD8 | 1:100 | Mouse monoclonal | Abcam |
| CD163 | 1:100 | Mouse monoclonal | Abcam |
| Foxp3 | 1:100 | Rabbit monoclonal | Abcam |
| PD1 | 1:100 | Rabbit monoclonal | Cell signaling technology |
| PD-L1 | 1:100 | Rabbit monoclonal | Cell signaling technology |
| OX-40 | 1:100 | Mouse monoclonal | Abcam |
| CK19 | 1:100 | Rabbit monoclonal | Abcam |
| CK7 | 1:100 | Rabbit monoclonal | Abcam |
| GPC3 | 1:100 | Rabbit monoclonal | Abcam |
| Hep-par1 | 1:100 | Rabbit monoclonal | Abcam |
