# Supplementary material for: The distribution of immune cells within combined hepatocellular carcinoma and cholangiocarcinoma predicts clinical outcome
Source: Clin Transl Med. 2020 Apr 18;10(1):45–56. doi: 10.1002/ctm2.11 (PMC7239312; doi:10.1002/ctm2.11)
Supplement: Supplementary file 5 — Supporting information [file CTM2-10-45-s005.docx]

**Table S2 The prognostic significance of the immune variables across different subregions.**

|  | | **Median overall survival**  **（Months）** | ***P* value** |
| --- | --- | --- | --- |
| **CD3** | | | |
| HCC component | Low(n=39) | 28.507 | **0.048** |
|  | High(n=17) | 47.289 |  |
| ICC component | Low(n=46) | 38.623 | 0.270 |
|  | High(n=10) | 28.686 |  |
| HCC IM | Low(n=31) | 28.202 | 0.069 |
|  | High(n=25) | 49.759 |  |
| ICC IM | Low(n=10) | 19.700 | **0.005** |
|  | High(n=46) | 42.846 |  |
| Peritumor liver | Low(n=18) | 24.995 | **0.009** |
|  | High(n=38) | 48.772 |  |
| **CD8** | | | |
| HCC component | Low(n=13) | 19.444 | **0.001** |
|  | High(n=43) | 44.305 |  |
| ICC component | Low(n=18) | 35.542 | 0.366 |
|  | High(n=38) | 38.597 |  |
| HCC IM | Low(n=43) | 34.729 | 0.060 |
|  | High(n=13) | 49.244 |  |
| ICC IM | Low(n=25) | 32.667 | **0.048** |
|  | High(n=31) | 40.156 |  |
| Peritumor liver | Low(n=28) | 24.832 | **0.004** |
|  | High(n=28) | 50.850 |  |
| **CD163** | | | |
| HCC component | Low(n=46) | 43.110 | **0.014** |
|  | High(n=10) | 16.215 |  |
| ICC component | Low(n=40) | 44.875 | **0.003** |
|  | High(n=16) | 26.876 |  |
| HCC IM | Low(n=10) | 45.466 | **<0.001** |
|  | High(n=45) | 18.797 |  |
| ICC IM | Low(n=32) | 37.867 | 0.933 |
|  | High(n=24) | 37.075 |  |
| Peritumor liver | Low(n=20) | 29.326 | 0.915 |
|  | High(n=36) | 40.811 |  |
| **Foxp3** | | | |
| HCC component | Low(n=43) | 44.984 | **0.021** |
|  | High(n=13) | 22.698 |  |
| ICC component | Low(n=38) | 44.944 | **0.033** |
|  | High(n=18) | 24.467 |  |
| HCC IM | Low(n=12) | 33.183 | 0.053 |
|  | High(n=44) | 36.043 |  |
| ICC IM | Low(n=34) | 36.077 | 0.594 |
|  | High(n=22) | 39.639 |  |
| Peritumor liver | Low(n=18) | 39.585 | 0.214 |
|  | High(n=38) | 32.038 |  |
| **PD1** | | | |
| HCC component | Low(n=41) | 28.635 | **0.024** |
|  | High(n=15) | 44.369 |  |
| ICC component | Low(n=11) | 37.717 | 0.230 |
|  | High(n=45) | 37.215 |  |
| HCC IM | Low(n=46) | 25.307 | **0.032** |
|  | High(n=10) | 42.187 |  |
| ICC IM | Low(n=42) | 21.074 | **0.029** |
|  | High(n=14) | 43.051 |  |
| Peritumor | Low(n=39) | 42.391 | 0.521 |
|  | High(n=17) | 29.546 |  |
| **OX-40** | | | |
| HCC component | Low(n=32) | 31.180 | 0.500 |
|  | High(n=24) | 40.502 |  |
| ICC component | Low(n=36) | 30.315 | 0.746 |
|  | High(n=20) | 39.897 |  |
| HCC IM | Low(n=29) | 28.727 | 0.544 |
|  | High(n=27) | 42.750 |  |
| ICC IM | Low(n=34) | 29.791 | 0.953 |
|  | High(n=22) | 41.307 |  |
| Peritumor liver | Low(n=48) | 38.692 | 0.257 |
|  | High(n=8) | 45.249 |  |
| **PD-L1** | | | |
| HCC component | Low(n=38) | 46.328 | **0.011** |
|  | High(n=18) | 23.412 |  |
| ICC component | Low(n=32) | 48.624 | **0.004** |
|  | High(n=24) | 24.455 |  |
| HCC IM | Low(n=27) | 48.681 | 0.111 |
|  | High(n=29) | 27.786 |  |
| ICC IM | Low(n=31) | 47.143 | 0.207 |
|  | High(n=25) | 28.063 |  |
| Peritumor liver | Low(n=43) | 41.822 | 0.130 |
|  | High(n=13) | 29.547 |  |

Kaplan-Meier analysis. *Abbreviation: IM: invasive margin.*
