# Supplementary material for: The distribution of immune cells within combined hepatocellular carcinoma and cholangiocarcinoma predicts clinical outcome
Source: Clin Transl Med. 2020 Apr 18;10(1):45–56. doi: 10.1002/ctm2.11 (PMC7239312; doi:10.1002/ctm2.11)
Supplement: Supplementary file 6 — Supporting information [file CTM2-10-45-s006.docx]

**Table S3. Correlation between the immune variables and clinicopathological features**

| **Variables** | **Cluster** | | | **Immune score** | | |
| --- | --- | --- | --- | --- | --- | --- |
|  | **Cluster1** | **Cluster2** | ***P*** | **High** | **Low** | ***P*** |
| **Age, year** |  |  |  |  |  |  |
| ≤60 | 12 | 25 | 0.948 | 26 | 11 | 0.338 |
| >60 | 6 | 13 |  | 16 | 3 |  |
| **Gender** |  |  |  |  |  |  |
| Male | 4 | 7 | 0.732 | 8 | 3 | 0.846 |
| Female | 14 | 31 |  | 34 | 11 |  |
| **Liver Cirrhosis** | | | | | | |
| No | 3 | 10 | 0.514 | 9 | 4 | 0.717 |
| Yes | 15 | 28 |  | 33 | 10 |  |
| **Max tumor size, cm** |  |  |  |  |  |  |
| ≤5 | 15 | 24 | **0.029** | 34 | 5 | **0.003** |
| ≥5 | 3 | 14 |  | 8 | 9 |  |
| **Tumor number** |  |  |  |  |  |  |
| Single | 10 | 29 | 0.682 | 37 | 9 | 0.999 |
| Multiple | 8 | 9 |  | 5 | 5 |  |
| **Microvascular invasion** |  |  |  |  |  |  |
| Absent | 15 | 29 | 0.999 | 33 | 11 | 0.999 |
| Present | 3 | 9 |  | 9 | 3 |  |
| **HbsAg** |  |  |  |  |  |  |
| Negative | 14 | 1 | **0.021** | 7 | 8 | **0.037** |
| Positive | 24 | 17 |  | 7 | 34 |  |
| **CA19-9,ng/ml** |  |  |  |  |  |  |
| ≤37 | 15 | 26 | 0.147 | 31 | 10 | 0.736 |
| ≥37 | 3 | 11 |  | 10 | 4 |  |
| **AFP, ng/ml** |  |  |  |  |  |  |
| ≤20 | 9 | 14 | 0.198 | 19 | 4 | 0.350 |
| ≥20 | 9 | 24 |  | 22 | 10 |  |
| **Pathological type** | | | | | | |
| Separated | 0 | 1 | 0.360 | 1 | 0 | 0.612 |
| Combined | 10 | 14 |  | 18 | 6 |  |
| Mixed type | 8 | 23 |  | 22 | 4 |  |
| **TNM stage** |  |  |  |  |  |  |
| Ia | 16 | 18 | **0.002** | 33 | 2 | **P<0.001** |
| Ib+II+IV | 2 | 20 |  | 9 | 12 |  |

Chi-squared test or Fisher’s exact test. *Abbreviation: TNM stage: tumor node metastasis stage; HbsAg: hepatitis B surface antigen; CA19-9:* *Carbohydrate Antigen 19-9; AFP:* *alpha fetoprotein.*
